# Deep Learning Models: Their Relationship with Embryonic Euploidies and Reproductive Outcomes

**DOI:** 10.3390/genes16080981

**Published:** 2025-08-20

**Authors:** Aikaterini Selntigia, Lucia Maresca, Diletta Montanino Oliva, Camilla Coianiz, Daniela Galliano

**Affiliations:** IVIRMA Global Research Alliance, IVIRMA Roma, 00161 Rome, Italy; lucia.maresca@ivirma.com (L.M.); diletta.montanino@ivirma.com (D.M.O.); camilla.coianiz@ivirma.com (C.C.); daniela.galliano@ivirma.com (D.G.)

**Keywords:** iDAScore, deep learning, euploidy, live birth rate

## Abstract

**Background:** Embryo selection in in vitro fertilization (IVF) aims to prioritize embryos with the highest reproductive potential. While preimplantation genetic testing for aneuploidy (PGT-A) remains the gold standard for identifying euploid embryos, it is invasive and not universally applicable. Deep learning (DL)-based models, such as the intelligent data analysis (iDA) score, have emerged as non-invasive alternatives for embryo assessment. This review critically evaluates the relationship between iDAScore (versions 1.0 and 2.0), embryo euploidy, and clinical outcomes, including live birth and miscarriage rates. **Methods:** A narrative review was performed using PubMed and Google Scholar, covering studies published from January 2020 to May 2025. The search included terms such as “iDAScore,” “deep learning,” “euploidy,” and “live birth.” Only English-language full-text studies assessing the predictive performance of iDAScore relative to chromosomal status or reproductive outcomes were included. **Results:** Six retrospective studies met the inclusion criteria. All reported a statistically significant association between higher iDAScore values and embryo euploidy. AUC values for euploidy prediction ranged from 0.60 to 0.68. In several studies, iDAScore was also positively associated with live birth rates and negatively with miscarriage rates. However, the predictive accuracy was moderate when restricted to euploid embryo cohorts, indicating that iDAScore may be more effective in broader populations where chromosomal status is unknown. **Conclusions:** iDAScore represents a promising adjunct to traditional embryo assessment. Although it cannot replace PGT-A, it may aid in embryo prioritization when genetic testing is not feasible. Larger prospective studies are warranted to further validate its clinical utility.

## 1. Introduction

The selection of the most viable embryo remains a central challenge in in vitro fertilization (IVF), with the ultimate goal of maximizing the chances of a healthy and full-term pregnancy. Traditionally, embryo selection has relied on morphological assessment and, more recently, on time-lapse imaging (TLI) and preimplantation genetic testing for aneuploidy (PGT-A). While PGT-A provides a reliable method for identifying euploid embryos, it is invasive, costly, and not always accessible or indicated for all patient populations.

In recent years, the use of TLI combined with artificial intelligence (AI) algorithms has emerged as a promising non-invasive tool for embryo evaluation. By refining embryo selection, an increase in the clinical pregnancy rate and a reduction in the time to pregnancy were observed [1].

Deep learning, a subset of machine learning within the broader field of AI, offers enhanced capabilities for embryo classification and promotes standardization in embryo selection within assisted reproductive technology (ART) laboratories. Unlike conventional methods, deep learning models—particularly those based on convolutional neural networks (CNNs)—can learn directly from image data, significantly improving prediction accuracy [2].

Several AI-based embryo scoring systems have been developed in recent years, each employing distinct algorithmic architectures, input data, and predictive endpoints. These include KIDScore D5/D6 (Vitrolife), ERICA (Embryo Ranking Intelligent Classification Algorithm, Igenomix), STORK/STORK-A (Weill Cornell Medicine), Life Whisperer (Presagen), EmbryoRanking, and CHLOE-EQ (Fairtility). While some of these tools focus on estimating implantation potential from time-lapse morphokinetic data, others aim to predict embryo ploidy from static images. Collectively, they illustrate the expanding role of AI in supporting embryo selection in ART [3,4,5].

The software “intelligent data analysis (iDA)” (Vitrolife A/S), which can be directly integrated into an EmbryoScope+ incubator (Vitrolife A/S), is an AI-based model trained on extensive time-lapse video datasets of embryos with known clinical outcomes. The system enables the assignment of scores that reflect the likelihood of implantation and live birth. It applies a deep learning algorithm to time-lapse videos, assigning scores from 1.0 to 9.9 based on developmental patterns [6]. iDAScore has been proposed as a means of predicting implantation potential [7]. Several studies conducted on embryos selected through standard clinical protocols have shown that iDAScore is correlated not only with blastocyst morphology and morphokinetic parameters [8] but also with embryo ploidy status [6]. The clinical relevance of these tools, however, remains under investigation, particularly in terms of their ability to serve as a substitute for PGT-A in predicting euploidy. Both versions of iDAScore (v1.0 and v2.0) have been evaluated in various studies, often with differing methodologies, patient populations, and outcome measures. As a result, the degree to which these deep learning models can accurately predict embryonic aneuploidy remains unclear [5,9,10,11,12,13,14].

The primary objective of this review was to synthesize and critically appraise the current evidence regarding the association between deep learning-based embryo assessment tools—specifically iDAScore v1.0 and v2.0—and embryo ploidy status, as well as reproductive outcomes, including live birth and miscarriage rates.

## 2. Materials and Methods

This narrative review aimed to explore recent evidence on the relationship between deep learning-based embryo evaluation tools and embryo euploidy, with or without the application of PGT-A and reproductive outcomes. The focus was on studies assessing the performance of iDAScore versions 1.0 and 2.0.

A literature search was conducted in PubMed, the primary biomedical database in reproductive medicine, and Google Scholar, which was included to capture the grey literature and recent publications not yet indexed in PubMed. Other databases, namely, Embase, Web of Science, and Scopus, were not included, as preliminary scoping searches indicated substantial overlap with PubMed for this specific topic, with no additional high-quality studies identified exclusively in those sources.

The search was restricted to articles published between January 2020 and May 2025, a period marked by significant developments in AI-driven embryo evaluation technologies. Only English-language full-text articles were included, defined as complete peer-reviewed publications available in their entirety. Preclinical studies, reviews, and abstracts from scientific meetings were excluded. When multiple publications reported results from the same patient cohort or dataset, only the most recent (January 2020 and May 2025) was included to avoid duplication of data.

The search terms employed were (iDAScore) AND (euploidy); (iDAScore) AND (live birth); (iDAScore) AND (embryo transfer).

Given the limited number of studies specifically evaluating iDAScore in relation to embryo euploidy, live birth, or implantation outcomes, we used focused search terms such as “iDAScore” combined with relevant endpoints (e.g., “euploidy,” “live birth,” “embryo transfer”). While broader AI-related terms could have identified additional studies, we prioritized specificity to capture the directly relevant literature and avoid inclusion of heterogeneous algorithms with different outcomes.

All included studies were based on published data; some studies may have involved Vitrolife, the manufacturer of iDAScore, in algorithm development or access to proprietary datasets. Our review is purely narrative.

## 3. Results

**Figure 1:**
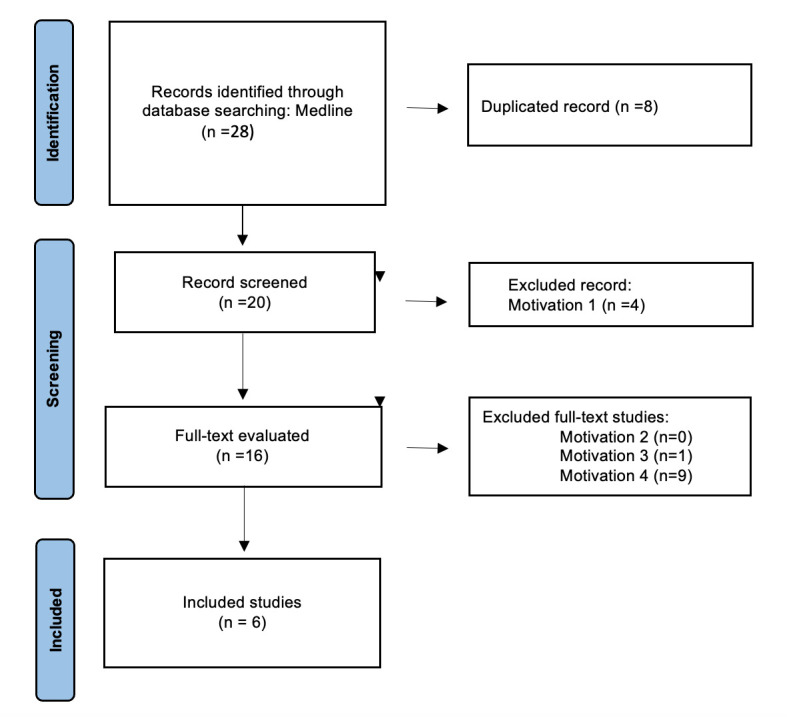
Flow diagram of the study identification and selection.

The article selection process is depicted in Figure 1. A total of 28 records were initially identified through electronic searches using the keywords specified above. Following the application of exclusion criteria, six studies were included in this review.

Multiple studies have investigated the predictive value of iDAScore and other AI-based embryo evaluation tools with a particular focus on their association with embryo euploidy. Across both retrospective and prospective studies, consistent evidence has emerged supporting the clinical relevance of iDAScore as a non-invasive method for prioritizing embryos with a higher likelihood of chromosomal normality. Table 1 shows the studies included in this review.

Ma et al. conducted a retrospective study to evaluate the predictive capacity of iDAScore v1.0 for identifying euploid embryos [9]. The analysis included a total of 3448 blastocysts made in 979 TL-PGT cycles, all of which were cultured in time-lapse incubators and subsequently analyzed through PGT-A. Each embryo was retrospectively assigned an iDAScore ranging from 1.0 to 9.9, based on morphokinetic parameters and morphological features extracted automatically from time-lapse videos, without the need for manual annotation.

The authors reported a statistically significant positive correlation between iDAScore and embryo euploidy status. Specifically, euploid embryos exhibited higher mean iDAScore values compared to aneuploid embryos. This association suggests that the algorithm is able to capture subtle developmental features that are positively linked to chromosomal normality, despite not being explicitly trained to predict ploidy. The discriminative ability of iDAScore alone was modest, with an area under the curve (AUC) of 0.612, indicating performance above chance but insufficient for diagnostic use. However, when combined with clinical and embryonic parameters, the AUC slightly increased to 0.688, suggesting a low additive value in embryo selection.

These results support the potential utility of iDAScore as a non-invasive tool for prioritizing embryos more likely to be chromosomally normal, particularly in settings where PGT-A is not routinely applied. Indeed, this finding underscores the model’s potential as a non-invasive proxy marker for embryo competence, particularly in settings where genetic testing may not be accessible or routinely performed. The study contributes to the growing body of evidence supporting the role of AI in refining embryo evaluation and confirms that AI-derived morphokinetic profiles may reflect underlying genomic integrity [9].

In line with these findings, Lee et al. explored whether iDAScore v1.0 could also serve as a predictor of clinical outcomes—specifically live birth rates—in embryos that had already undergone PGT-A. In their study, 482 single blastocyst transfers involving either euploid or mosaic embryos were retrospectively analyzed. The authors observed that embryos with an iDAScore value ≤ 7.8 were associated with significantly lower live birth rates and a higher rate of miscarriage compared to those with higher scores. This observation highlights the potential of iDAScore not only as a tool for embryo prioritization in the absence of genetic testing, but also as a means of refining selection even within a genetically screened population. Notably, the study emphasized that iDAScore should be regarded as a complementary tool rather than a substitute for PGT-A, as it does not directly assess chromosomal status. Its strength lies in capturing dynamic developmental characteristics that may influence implantation potential, thereby adding another dimension to embryo selection strategies aimed at optimizing clinical outcomes [10].

Cimadomo et al. conducted a retrospective multicenter study aimed at evaluating the performance of iDAScore v1.0, a deep learning-based embryo ranking algorithm, during IVF cycles with PGT-A [11]. The algorithm was retrospectively applied to a large dataset of blastocysts cultured in time-lapse incubators and previously analyzed for chromosomal status.

The study found that iDAScore was significantly correlated with conventional morphological features, such as inner cell mass and trophectoderm quality, as well as with the day of blastocyst expansion. Embryos with earlier development and better morphology consistently received higher scores, confirming that the algorithm aligns with established embryological indicators of embryo quality.

In terms of chromosomal status, euploid embryos displayed higher iDAScore values compared to aneuploid ones. Although the association was statistically significant, the score’s ability to discriminate euploidy remained moderate, with an AUC of 0.60. This suggests that while iDAScore captures aspects related to developmental competence, it cannot replace genetic testing.

Interestingly, iDAScore also showed a positive association with clinical outcomes, such as live births following euploid single embryo transfers (AUC of 0.66). Its predictive performance in this context was comparable to that of embryologists’ assessments, but with the advantage of being objective and reproducible.

Simulation analyses have further highlighted the potential utility of iDAScore in embryo selection. When applied to cohorts containing both euploid and aneuploid embryos, the algorithm more frequently ranked euploid embryos as top-quality compared to traditional morphological evaluation. In cases with multiple euploid embryos, iDAScore prioritized the embryo leading to a live birth in a notable proportion of cases, suggesting a possible role in refining transfer decisions even when genetic data are available. Overall, the study supports the integration of AI tools like iDAScore as complementary decision-support systems for embryo selection, particularly useful in settings where PGT-A is not accessible or applicable [11].

In a large-scale multicenter study of iDAScore v2.0, Theilgaard Lassen et al. provided evidence of a relationship between the model’s predictions and embryo chromosomal status [12]. Specifically, a significantly lower AUC (0.516) for implantation prediction was observed in Clinic 21 compared to other centers. This clinic was the only one to routinely apply PGT-A, thus transferring exclusively euploid embryos. The authors interpreted this performance drop as a consequence of the reduced biological variability in the transferred cohort, which limited the model’s ability to discriminate between embryos with different implantation potentials. To further investigate this aspect, they tested iDAScore v2.0 on a dataset of embryos from Clinic 21 with known ploidy status, revealing an AUC of 0.68 in distinguishing euploid from aneuploid embryos. This result supports the view that the model’s output is influenced, at least in part, by features that are systematically associated with embryo ploidy. Such findings contribute to the growing body of evidence that deep learning-based embryo assessment tools may reflect underlying chromosomal competence [12].

Similarly, Bori et al. evaluated the relationship between iDAScore v2.0 and embryo ploidy status in a large retrospective analysis involving 7082 embryos with known PGT-A results. The study confirmed a significant association between iDAScore and chromosomal normality: euploid embryos had a higher mean score than aneuploid ones (6.3 ± 2.5 vs. 5.1 ± 2.6; *p* < 0.001). Furthermore, the proportion of euploid embryos increased progressively with rising score values, suggesting that higher iDAScore intervals were enriched for chromosomally normal embryos. This association was consistent across maternal age groups and morphological categories, supporting the robustness of the observation. In an additional cohort of non-PGT-A cycles, the authors also found that embryos leading to miscarriage had significantly lower average iDAScore values compared to those resulting in live births (6.6 ± 2.3 vs. 6.9 ± 2.2; *p* < 0.001). Although a clear linear trend in miscarriage rates across score groups was not observed, these findings reinforce the potential of iDAScore to serve as a non-invasive indicator of embryo competence, with implications both for prioritizing euploid embryos and for refining selection among genetically untested embryos [13].

Although the study by Ueno et al. did not directly assess embryo ploidy status, it demonstrated a significant correlation between higher iDAScore v1.0 values and increased live birth rates, as well as decreased miscarriage rates [14]. Given that euploid embryos are known to have a lower risk of miscarriage [15] and result in live births, these findings provide indirect clinical support for a possible association between iDAScore and embryo chromosomal normality. This reinforces the biological plausibility of a link between higher iDAScore values and euploid status.

## 4. Discussion

Although the number of studies investigating the association between iDAScore and embryo euploidy remains limited, emerging evidence suggests a positive correlation between higher iDAScore values and the likelihood of positive reproductive outcomes in terms of a higher chance of live birth rates. This indicates a potential role for deep learning-based assessment tools in supporting embryo selection. However, the current body of literature is still insufficient to draw definitive conclusions, and further research—particularly large-scale, multicenter studies—is needed to validate these preliminary findings and establish more robust clinical guidelines.

To date, many of the available studies report an AUC of approximately 0.6–0.7 [9,10,11,12,13,14], indicating a moderate level of sensitivity and predictive reliability. This performance suggests that while iDAScore (both version 1.0 and version 2.0) may contribute useful information, it cannot replace PGT-A as a stand-alone method for assessing chromosomal status.

Nonetheless, iDAScore may have clinical utility in specific scenarios where PGT-A is not feasible or not indicated. For instance, it could serve as an additional tool in the management of patients with idiopathic infertility, particularly in women under the age of 35, as well as in cases where PGT-A is not accessible due to economic constraints, or in patients undergoing treatment with donor oocytes, where genetic testing of the embryo may not be routinely performed. In such contexts, the use of AI-based scoring systems could aid in optimizing embryo selection while avoiding invasive procedures.

As reported by Ueno et al., iDAScore has been shown to be significantly associated with live birth rates, suggesting a potential role in predicting successful outcomes [14].

Nonetheless, Theilgaard Lassen et al. demonstrated that the predictive value of iDAScore is only clinically relevant when applied to cohorts of embryos with unknown ploidy status. When restricted to euploid embryos, iDAScore’s association with implantation potential becomes negligible, as reflected by an AUC of 0.516. This finding suggests that iDAScore may primarily capture morphological or developmental features that correlate with aneuploidy risk, rather than serving as an independent predictor of implantation among chromosomally normal embryos [12].

Future developments of the iDAScore algorithm may enhance its predictive performance even within cohorts of euploid embryos, potentially allowing it to contribute more meaningfully to embryo selection in settings where genetic screening has already been performed.

It is important to note that, among the studies reviewed, only Ueno et al. evaluated iDAScore in a non-PGT-A setting, focusing on clinical outcomes rather than embryo chromosomal status. All other available data on the predictive value of iDAScore for euploidy are derived from cohorts in which PGT-A was performed, thereby allowing chromosomal status to be directly ascertained. Consequently, the utility of iDAScore in predicting euploidy is currently validated exclusively in PGT-A cohorts. Extrapolating these findings to non-PGT-A populations should therefore be approached with caution, as the endpoints assessed—such as live birth or implantation rates—reflect broader clinical performance rather than direct euploidy prediction. Prospective, large-scale studies specifically conducted in non-PGT-A populations are needed to determine whether iDAScore can provide reliable guidance for embryo selection in settings where genetic testing is not performed.

It should be noted that the current evidence on deep learning-based embryo evaluation tools is largely derived from retrospective studies. The absence of prospective or randomized controlled trials limits the strength of the conclusions that can be drawn. Nevertheless, this narrative review aims to provide a critical synthesis of emerging data and highlight areas where high-quality prospective research is needed to validate the clinical utility of AI-based embryo assessment. An additional limitation of the available literature is the large discrepancy in sample sizes across studies, ranging from a few thousand to several hundred thousand embryos. Such variability may influence the robustness of statistical estimates and contribute to heterogeneity in reported outcomes. As this is a narrative review, no formal weighting or meta-analytic adjustment was performed; therefore, the findings should be interpreted with caution, and future research with standardized methodologies and comparable sample sizes is warranted. A further limitation relates to the proprietary nature of the iDAScore algorithm. Full details on the composition of the training datasets and the specific architecture used are not publicly disclosed, which prevents independent evaluation of the model’s generalizability and potential risk of overfitting. The available literature provides only partial methodological descriptions; therefore, our synthesis is based solely on information reported in peer-reviewed studies and manufacturer documentation. Furthermore, a performance drop in an all-euploid cohort suggests that iDAScore may, to some extent, capture features that correlate with ploidy status rather than directly assessing implantation competence. Such a dependency could represent a form of model bias, whereby predictive accuracy is partly driven by underlying chromosomal variability in the training data. Consequently, in populations with reduced ploidy variability, the algorithm’s discriminative power may diminish. Future research should aim to disentangle ploidy-related features from those strictly associated with implantation potential, possibly through training datasets enriched with euploid-only embryos from diverse clinical settings.

It should also be noted that this review does not aim to determine whether version 1.0 or 2.0 of iDAScore is more effective in predicting embryo euploidy, as comparative studies on this topic are still limited and inconclusive. While some studies have reported improved performance with iDAScore v2.0 over v1.0, particularly in predicting implantation and live birth outcomes, the evidence regarding their relative effectiveness in predicting embryo euploidy remains insufficient. An important consideration for future work is the interpretability of AI-based embryo assessment tools. While iDAScore provides predictive scores for implantation and euploidy, the specific morphological or developmental features driving these predictions are not fully elucidated. Enhancing model transparency and establishing the biological plausibility of its features will be essential to increase clinician trust and support broader clinical adoption. Further research, especially large-scale, multicenter studies, is needed to validate these preliminary findings and establish more robust clinical guidelines.

## 5. Conclusions

In conclusion, current evidence suggests that iDAScore could complement conventional embryo assessment strategies. However, its current predictive value for euploidy remains limited, and further validation is essential before its broader clinical application can be recommended. The integration of deep learning tools into ART must be approached with caution, ensuring that patient care remains evidence-based and individualized.

Integrating morphological, genetic, and AI-based assessments could, in theory, support a more personalized embryo selection process, potentially improving the efficiency and success rates of ART. However, while this integrative approach appears promising, its clinical applicability remains limited at present. Further large-scale, well-designed randomized controlled trials are warranted to confirm and provide more definitive evidence of these findings.

## Figures and Tables

**Table 1:** Summary of recent studies evaluating the concordance between iDAScore v1.0/v2.0 and embryo euploidy and the association between iDAScore v1.0/v2.0 and reproductive outcomes. Preimplantation genetic testing for aneuploidy (PGT-A); intelligent data analysis (iDA); not applicabile (N/A).

Study	Type of Study	N	Tools	AUC Euploidy	CI 95%	*p*-Value	Live Birth Rate	Miscarriage Rate
Ma et al., 2024 [9]	Retrospective	3448	iDAScore v1.0 + PGT-A	0.612	0.796–0.887	<0.001	N/A	N/A
Lee et al., 2024 [10]	Retrospective	482	iDAScore v1.0 + PGT-A	0.67	0.62–0.72	<0.001	Positively associated	Negative associated
Cimadomo et al., 2023 [11]	Retrospective multicenter	3604	iDAScore v1.0 + PGT-A	0.60	0.59–0.62	<0.01	Positively associated	N/A
Theilgaard Lassen et al., 2023 [12]	Retrospective multicentric	249.635	iDAScore v2.0 + PGT-A	0.68	N/A	<0.001	N/A	N/A
Bori et al., 2025 [13]	Retrospective	70.456	iDAScore v2.0 + PGT-A	0.635	1.14–1.21	<0.001	Positively associated	Negatively associated
Ueno et al., 2022 [14]	Retrospective	3010	iDAScore v1.0	N/A	1.666–1.976	<0.05	Positively associated	Negatively associated

## References

[1] Meseguer M., Rubio I., Cruz M., Basile N., Marcos J., Requena A. (2012). Embryo incubation and selection in a time-lapse monitoring system improves pregnancy outcome compared with a standard incubator: A retrospective cohort study. Fertil. Steril..

[2] Thirumalaraju P., Kanakasabapathy M.K., Bormann C.L., Gupta R., Pooniwala R., Kandula H., Souter I., Dimitriadis I., Shafiee H. (2020). Evaluation of Deep Convolutional Neural Networks in Classifying Human Embryo Images Based on Their Morphological Quality. arXiv.

[3] Chavez-Badiola A., Flores-Saiffe-Farías A., Mendizabal-Ruiz G., Drakeley A.J., Cohen J. (2020). Embryo Ranking Intelligent Classification Algorithm (ERICA): Artificial intelligence clinical assistant predicting embryo ploidy and implantation. Reprod. Biomed. Online.

[4] Gazzo E., Peña F., Valdéz F., Chung A., Bonomini C., Ascenzo M., Velit M., Escudero E. (2020). The KidscoreTM D5 algorithm as an additional tool to morphological assessment and PGT-A in embryo selection: A time-lapse study. JBRA Assist. Reprod..

[5] Kato K., Ueno S., Berntsen J., Kragh M.F., Okimura T., Kuroda T. (2023). Does embryo categorization by existing artificial intelligence, morphokinetic or morphological embryo selection models correlate with blastocyst euploidy rates?. Reprod. Biomed. Online.

[6] Vitrolife iDAScore-Intelligent Data Analysis for Embryo Evaluation. https://www.vitrolife.com/our-products/idascore-intelligent-data-analysis-for-embryo-evaluation/.

[7] Sarandi S., Boumerdassi Y., O’Neill L., Puy V., Sifer C. (2023). Intérêt de l’iDAScore (intelligent Data Analysis Score) dans la pratique quotidienne d’un laboratoire de FIV pour la sélection embryonnaire: Résultats d’une étude préliminaire [Interest of iDAScore (intelligent Data Analysis Score) for Embryo Selection in Routine IVF Laboratory Practice: Results of a Preliminary Study]. Gynecol. Obstet. Fertil. Senol..

[8] Ezoe K., Shimazaki K., Miki T., Takahashi T., Tanimura Y., Amagai A., Sawado A., Akaike H., Mogi M., Kaneko S. (2022). Association between a deep learning-based scoring system with morphokinetics and morphological alterations in human embryos. Reprod. Biomed. Online.

[9] Ma B.X., Zhao G.N., Yi Z.F., Yang Y.L., Jin L., Huang B. (2024). Enhancing clinical utility: Deep learning-based embryo scoring model for non-invasive aneuploidy prediction. Reprod. Biol. Endocrinol..

[10] Lee C.I., Huang C.C., Lee T.H., Chen H.H., Cheng E.H., Lin P.Y., Yu T.N., Chen C.I., Chen C.H., Lee M.S. (2024). Associations between the artificial intelligence scoring system and live birth outcomes in preimplantation genetic testing for aneuploidy cycles. Reprod. Biol. Endocrinol..

[11] Cimadomo D., Chiappetta V., Innocenti F., Saturno G., Taggi M., Marconetto A., Casciani V., Albricci L., Maggiulli R., Coticchio G. (2023). Towards automation in IVF: Pre-clinical validation of a deep learning-based embryo grading system during PGT-A cycles. J. Clin. Med..

[12] Theilgaard Lassen J., Fly Kragh M., Rimestad J., Nygård Johansen M., Berntsen J. (2023). Development and validation of deep learning-based embryo selection across multiple days of transfer. Sci. Rep..

[13] Bori L., Toschi M., Esteve R., Delgado A., Pellicer A., Meseguer M. (2025). External validation of a fully automated evaluation tool: A retrospective analysis of 68,471 scored embryos. Fertil. Steril..

[14] Ueno S., Berntsen J., Ito M., Okimura T., Kato K. (2022). Correlation between an annotation-free embryo scoring system based on deep learning and live birth/neonatal outcomes after single vitrified-warmed blastocyst transfer: A single-centre, large-cohort retrospective study. J. Assist. Reprod. Genet..

[15] Simopoulou M., Sfakianoudis K., Maziotis E., Tsioulou P., Grigoriadis S., Rapani A., Giannelou P., Asimakopoulou M., Kokkali G., Pantou A. (2021). PGT-A: Who and when? A systematic review and network meta-analysis of RCTs. J. Assist. Reprod. Genet..

